# Design of embodied interfaces for engaging spatial cognition

**DOI:** 10.1186/s41235-016-0032-5

**Published:** 2016-12-07

**Authors:** Paul G. Clifton, Jack Shen-Kuen Chang, Georgina Yeboah, Alison Doucette, Sanjay Chandrasekharan, Michael Nitsche, Timothy Welsh, Ali Mazalek

**Affiliations:** 1 Georgia Institute of Technology, Atlanta, GA USA; 2 Purdue University, West Lafayette, IN USA; 3 University of Toronto, Toronto, ON Canada; 4 Tata Institute of Fundamental Research, Mumbai, Maharashtra India; 5 Ryerson University, Toronto, ON USA

**Keywords:** Embodied cognition, Tangible and embodied interaction, Spatial cognition, STEM, Interaction design

## Abstract

Aspects of spatial cognition, specifically spatial skills, are strongly correlated with interest and success in STEM courses and STEM-related professions. Because growth in STEM-related industries is expected to continue for the foreseeable future, it is important to develop evidence-based and theoretically grounded methods and interventions that can help train relevant spatial skills. In this article, we discuss research showing that aspects of spatial cognition are embodied and how these findings and theoretical developments can be used to influence the design of tangible and embodied interfaces (TEIs). TEIs seek to bring interaction with digital content off the screen and into the physical environment. By incorporating physical movement and tangible feedback in digital systems, TEIs can leverage the relationship between the body and spatial cognition to engage, support, or improve spatial skills. We use this knowledge to define a design space for TEIs that engage spatial cognition and illustrate how TEIs that are designed and evaluated from a spatial cognition perspective can expand the design space in ways that contribute to the fields of cognitive science and human computer interaction.

## Significance

The research and conceptualizations presented here integrate leading-edge developments to the fields of cognitive science and human computer interaction (HCI) and provide a new framework for future work. Through our work, we have developed a critical analysis of previously developed systems that highlights the relationships between body and spatial cognition that tangible and embodied interactive systems support. We have also developed our own content for tangible systems, illustrating how designing from a spatial cognition perspective can lead to new design and research opportunities. Our research and design process both informs the design of interactions with computational systems and leads to new knowledge about the relationships between the body, the digital medium, and cognition (in particular, spatial cognition).

The design space presented in this article illustrates the link between the way people interact with tangible and embodied interfaces (TEIs), the content the systems present, and the aspects of spatial cognition those systems engage. The design space classifies aspects of spatial cognition based on how they relate to the body and shows how a scale-based description of interactions is useful for linking interactions with spatial cognition. These classifications are useful for both cognitive science and human computer interaction researchers. Importantly, they lay out a matrix for future designs dealing with spatial cognition and also provide an analytical framework for the discussion of existing designs. Our own projects illustrate how designing digital systems from a spatial cognition perspective may lead to the creation of a strong sense of embodiment and, as a result, new ways to engage spatial cognition by leveraging the unique capabilities of digital media.

## Introduction

Between Super and Bachrach’s (1957) report to the meta-analysis of Wai, Lubinski, and Benbow (2009) of several longitudinal studies of student aptitude, success, and careers, there is nearly 60 years of research showing a strong relationship between success in STEM fields and spatial abilities (Lubinski & Benbow, 2006; Shea, Lubinski, & Benbow, 2001; Super & Bachrach, 1957; Wai et al., 2009; Webb, Lubinski, & Benbow, 2007). Spatial abilities, as they are discussed in those studies and defined by Lohman (1979), are “the ability to generate, retain, retrieve and transform well-structured visual images.” For example, mental rotation ability refers to the ability to construct a mental representation of an object and then draw some conclusions about that object after it has undergone some transformation. Mental rotation is just one of many spatial abilities that have been linked to success and pursuit of careers in STEM fields (Newcombe & Shipley, 2015).

This relationship between STEM and spatial abilities has led to calls from governments for research about how to support these skills in the education system (President’s Council of Advisors on Science and Technology, 2012; ServiceOntario Publications, 2014). Researchers have answered that call by generating new knowledge and creating research networks and centers like the *Spatial Intelligence and Learning Center* (SILC). Some researchers like Quarles and Wu have focused on creating tools that support the use of spatial skills in learning complex systems (e.g. Quarles, Lampotang, Fischler, Fishwick, & Lok, 2008; Wu et al., 2011). Other researchers have looked at which specific spatial skills are used in science disciplines, which could lead to new strategies about how best to support them (Resnick & Shipley, 2013). However, little research has been done to determine the best methods and approaches for improving spatial skills in general and, in particular, in students with low spatial ability scores, even though there is research showing that it may be possible (Uttal et al., 2013).

Recent research indicates that there is a link between the body, actions, and spatial cognition (Avraamides, Loomis, Klatzky, & Golledge, 2004; Boroditsky, 2000; Chandrasekharan, Athreya, & Srinivasan, 2006; Golledge, 1999; Lozano, Hard, & Tversky, 2007; May, 2004; Morsella & Krauss, 2004; Mou, McNamara, Valiquette, & Rump, 2004; Portugali, 1996; Taylor & Tversky, 1992; Tversky, 2000; Tversky & Hard, 2009). While the specific cognitive mechanisms underlying this link are still under debate, the fact that the body plays an active role in the cognition of space is, at this point, well-established. Because of the link between body, action, and space, it is likely that designing spatial-skill interventions that engage the body and action systems will be effective. The goal of our recent work, and the present paper, is to establish a framework for these interventions which can be used to inform the design of technologies that effectively train spatial skills.

Digital media has made it possible to create experiences of simulated objects and spaces that enable people to apply spatial skills in ways that would be impossible in typical real-world interactions (Benedikt, 1991; Murray, 1997; Nitsche, 2008). Digital media can also be used to create environments with spatial properties that are very different from the real world (Ambinder, Wang, Crowell, Francis, & Brinkmann, 2009; Kortemeyer, Tan, & Schirra, 2013). Practically speaking, digital media interfaces have tended to focus on the visual experience of a system’s output (e.g. high-definition and high-framerate graphics) rather than on creating input devices that engage the body and motor system. While user interfaces like windowed operating systems use spatial metaphors for their organizational structures, the physical connection to the space of infinitely nested folders is limited to pointing and clicking actions. Likewise, video games include sophisticated simulated environments and countless ways to interact with and traverse them, but the physical input experience is often limited to pressing buttons on a controller. Until recently, there has been a very limited relationship between the spaces of the digital content and the physical world. That is, the relatively arbitrary action–effect relationships established in these systems are quite simple and disconnected from the rich array of sensorimotor experiences with tight spatial relationships experienced in real life.

An evolving field of HCI, known as TEI, aims to bridge the gap between the physical environment and digital content. TEIs incorporate physical objects and sensing systems to give people ways to use their bodies to interact with digital information. An emerging trend in TEI research is how embodied interaction can leverage or be leveraged by embodied cognition research. In this paper, we describe the relationship between the embodied aspects of TEIs and the embodied aspects of spatial cognition.

In this paper, we review and present evidence that spatial cognition is shaped by bodily and action states and describe the field of tangible and embodied interfaces through related theoretical perspectives. We then define a design space that links TEIs with aspects of spatial cognition through the concept of scale. We then place different TEIs within the design space and discuss each of the systems with respect to how the content of the systems leverages the relationship between the body and spatial cognition for different purposes. We then show how designing TEIs from a spatial cognition perspective leads to new relationships between the body, digital content, and space. The paper closes with a discussion of the challenges we face in applying our research to STEM education and practice as well as the broader impacts of our work.

## Background

Our research draws from and integrates cognitive science research showing that spatial cognition is shaped by the body and action, and from design research focused on digital systems that incorporate tangible and embodied interactions.

### Spatial cognition is shaped by bodily and action states

Ultimately, perceptual and cognitive processes have developed through evolution to enable individuals to act in the environment and achieve a series of goals to survive. The ways in which individuals can achieve the goals, and indeed the goals themselves, are determined by the characteristics of the environment in which they are acting (e.g. steepness of hills, how far objects are apart) and action capabilities of the body (e.g. healthy, fatigued, or hungry). Tremendous research efforts have been dedicated to understanding how sensory information about the body and the environment is converted in action. Some of these recent studies of spatial abilities, perception, and navigation have provided evidence for the ways that spatial cognition and the body are related. This research has been shaped by and led to the development of two main approaches to understanding spatial and other cognitive processes. These two main approaches are ideomotor theory and ecological-based embodied cognition. Even though these approaches differ on the underlying mechanisms thought to support the interaction between the body, action, and cognition, both perspectives often lead to similar predictions on how the action system is tightly linked with and influences perception and cognition.

Ideomotor theory has emerged out of information processing and representational accounts of the series of events that occur from sensation, through perception and cognition, to action. According to information processing-based theories, perceptions, thoughts, and actions are represented and, importantly, stored in the brain by the activity of specific subsets of neurons. Earlier information processing models were based on the notion that sensory information is converted into actions through a series of processing stages that occur in a single direction (sensation–perception–cognition–action planning) because the representations activated in the subsequent stages were determined by the results of the preceding stages. Thus, although perception is connected to action, the influence is unidirectional with results of the perceptual processing helping to activate and shape actions, but not the reverse (i.e. action would not shape perception). Ideomotor theories (e.g. Hommel, Müsseler, Aschersleben, & Prinz, 2001) are based on the notion that perception and action systems are reciprocally interconnected and that, through experience, representations of specific sensory/perceptual events become tightly bound to the representations of specific actions. The result of the perception–action binding is a set of bidirectional interactions wherein the activation of a perceptual representation (via the reception of a specific series of sensations) can automatically prime the associated action, and conversely the activation of an action representation (via a decision and desire to move) can automatically activate the representation of the associated perceptual event. It is through this latter action-to-perception direction of activation that the action and bodily systems can prime or shape perceptual representations and processes.

The embodied approaches to cognition have largely evolved from ecological approaches to cognition (e.g. Gibson, 1966) which, in contrast to information processing theories, reject the notion of representations. Instead of action selection and planning being the result of a series of processing events relying on representations, action possibilities (affordances) are directly drawn (perceived) from the environment. Because the bodily and action status of the individual influences the types of actions the individual can perform, these factors influence the affordances and perception of the environment. In this way, perception and other cognitive events are embedded firmly in the body and the environment in which body is acting, with the consequence of perception and cognition being tightly linked to and shaped by the bodily and action state of the body.

In the case of spatial abilities, a growing series of studies have revealed that the action and bodily state of the individual influences the efficiency of these abilities. For example, a series of studies on mental rotation have shown that people perform congruent actions when solving complex mental rotation tasks, implying that spatial skills are scaffolded or enhanced by activation of the motor system (Chandrasekharan et al., 2006; Wohlschläger, 2001). Other studies have shown similar relationships between the body and spatial skills including perspective taking (Hegarty & Waller, 2004), scaling (Proffitt, 2013), and navigation and orientation (Darken, Peterson, & Orientation, 2001; Hegarty, Montello, Richardson, Ishikawa, & Lovelace, 2006). Similarly, perception of objects and spaces has been shown to be related to the state of the body. Proffitt and colleagues have shown, for example, that wearing a heavy backpack makes hills appear steeper (Bhalla & Proffitt, 1999) and distances appear longer (Witt, Proffitt, & Epstein, 2005). These effects are related to health and age (Bhalla & Proffitt, 1999) and skill (Taylor, Witt, & Sugovic, 2011; Witt & Proffitt, 2005). Finally, the body has been shown to play a role in the construction of mental representations of navigable spaces. Hegarty et al. (2006) showed that people who physically navigate an environment are better able to make judgments about the distances and directions between points of interest than people who navigated a virtual environment or watched a video of the environment being navigated. Overall, regardless of whether one prefers an ideomotor-based or ecological-based account of these findings, it is clear that the body and action history of the individual has been shown to influence the perception of space and the application of spatial skills.

### TEIs engage spatial cognition through the body and action

Given the relationships between the body, action, and space, it makes sense to design digital media-based interventions that engage spatial skills through physical movement. Digital technology has made it possible to create environments that engage spatial skills in novel ways. Because TEIs couple physical movements with the flexibility of digital technology, they are particularly well-suited for systems that engage and develop spatial skills.

Early TEIs used physical objects to represent digital data. For example, Durrel Bishop’s Marble Answering Machine links a digital recording of a voicemail with a physical marble (Poynor, 1995). To listen to the voicemail, a person places the marble in a particular spot on the machine. The machine recognizes the marble and plays the message. To delete the message, a person just puts the marble back in the machine’s hopper. Systems like the Marble Answering Machine make it possible for people to use the same skills they use in the physical world to engage with digital content. The “System descriptions” section later in the paper describes several tangible interfaces in detail, with respect to how they engage spatial cognition.

Extending this line of thinking has led to a wide variety of novel tangible interfaces and ways to think about them. Recently, researchers have become interested in the cognitive aspects of using the body to engage with digital content. Antle and Wang (2013) showed that a tangible puzzle interface leads to an increase in the use of epistemic actions. This study compared the motor-cognitive strategies people used when solving a jigsaw puzzle on a touch screen or when using a tangible interface that used the puzzle pieces as physical objects on an interactive tabletop (Antle & Wang, 2013). People using the tangible interface took less time to solve the puzzle, were more likely to sort the pieces, and made more movements—like rotating the pieces or testing placements. People use similar strategies when using tangible interfaces as when using traditional physical tools. Based on the current interest in embodied cognition within the TEI community, van Dijk, van der Lugt, and Hummels (2013) defined three “flavors” of embodied cognition and discussed how they relate to the design of TEI systems: distributed representation and computation (DRC), socially situated practice (SSP), and sensorimotor coupling and enactment (SCE). Of the three, SCE most directly relates to systems that specifically target spatial cognition. SCE refers to the ways that movement and perception inform each other and lead to behavior and cognition. However, there is little research from the field of TEI or from the cognitive sciences that addresses how SCE plays out in embodied digital systems:

*If we want to design systems that support the way people make sense of the world around them, the question is whether sensorimotor couplings are going to be enough, or whether sensorimotor theory is mostly useful when designing for ‘bodily phenomena’, like sports, or feelings of stress. That is, can sensorimotor theory help us get a grip on the more ‘cognitive’ activities for which we normally would use words like ‘remembering’, ‘thinking’, ‘representing’, ‘deciding’, ‘creativity’, ‘communication’, and so on?* (van Dijk et al., 2013).

Our research addresses this question directly by drawing design inspiration from known relationships between the body and spatial cognition and evaluating systems designed from this perspective using cognitive science methods. This research approach has led to the definition of the design space presented below and has informed our own design work, which has broadened our understanding of the relationships between body, action, and space from both design and cognitive science perspectives.

## Defining the design space

**Fig. 1:**
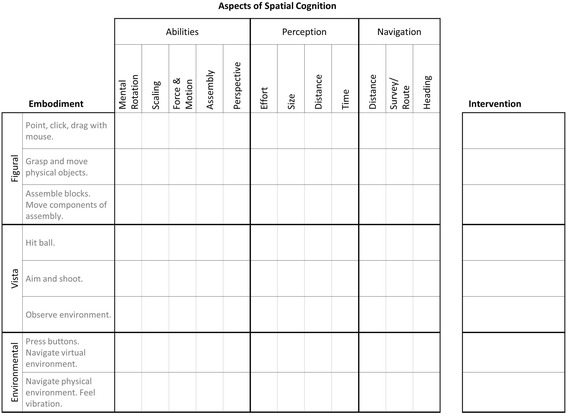
The design space defines embodied interfaces that engage spatial cognition in terms of the way that they engage the body (Embodiment), the aspect of spatial cognition they engage, and the spatial task that they ask a user to perform (Intervention)

Based on our analysis of existing TEIs, spatial cognition research, and our own experiences designing relevant systems, we identified three important elements of interactive systems that engage spatial cognition: the embodied aspect of the system (i.e. how the system engages the body and action systems), the aspect of spatial cognition the system engages, and the intervention that the system supports. With these elements in mind, the design space shown in Fig. 1 can be defined for digital systems that use a dynamic combination of embodiment and intervention to engage, support, or alter some aspect of spatial cognition.

The following sections describe embodiment, aspects of spatial cognition, and intervention and the different categories they contain.

### Embodiment

The “embodiment” aspect describes the ways that systems engage the body and action. The list shown in Fig. 1 is not, and cannot be, exhaustive. Any time a designer creates a new way for people to use their bodies to interact with technology, a new item could be added to the list. This particular set of ways that TEIs engage the body is drawn from our own analysis, which focused on TEIs that leverage embodiment as a way to engage spatial cognition.

Each method for engaging the body is classified based on a parameter-termed scale—figural, vista, or environmental—as defined by Montello (1993). Figural scale embodied interactions involve grasping and moving physical objects or controlling virtual objects as if they were real objects that could be manipulated using the hands. Vista scale embodied interactions create ways to engage with large content that is visible at a distance or alter the visible qualities of a vista scale space. Establishing embodiment at an environmental scale requires users to navigate a physical or virtual environment. The distinctions between and characteristics of these scales will be expanded upon and made more clear in a subsequent section on “System descriptions”.

### Aspects of spatial cognition

The “aspects of spatial cognition” axis lists the different aspects of spatial cognition that are engaged by tangible and embodied interaction systems. The categories—abilities, perception, and navigation—broadly group different aspects of spatial cognition. They are drawn from analysis of the different types of spatial cognition described in prior research (see http://www.silccenter.org/index.php/resources/testsainstruments for examples) and focus, in particular, on the skills that have been shown to relate to the body.

The abilities category contains spatial skills that relate to the performance of mental operations on images or objects. Spatial abilities typically relate to operations that could be performed on figural scale objects, such as rotation or assembly. For example, mental rotation is the ability to mentally represent an object and operate on that object. Mental rotation has been shown to be linked to the body with tests showing the effect of performing congruent and non-congruent actions when attempting to solve complicated mental rotation problems (Chandrasekharan et al., 2006). These findings lead to the idea that the motor system is leveraged in the use of mental rotation skills.

The perception category lists the elements of an environment and the objects it contains that are perceived differently depending on the state of the body. For example, a series of studies by Proffitt and colleagues have shown that wearing a heavy backpack, which would make it more difficult and effortful to move around, makes hills appear steeper (Bhalla & Proffitt, 1999) and distances appear longer (Witt et al., 2005). Such findings support the notion that the action potential of the body influences the perception of environmental characteristics.

Finally, the navigation category highlights the aspects of an environment or the qualities of a mental representation of that environment that are influenced by physical movement through that environment. For example, Hegarty, et al. reported that people who watched a video of someone else navigating an environment made poorer judgements about distances and directions than people who actually physically navigated the environment (Hegarty et al., 2006). This finding indicates that experience involving both perception and action in an environment enhances navigation of that space to a greater degree than experience involving perception alone.

### Intervention

Intervention is a descriptive column that is filled in with the content of the systems that will be plotted in the diagram. Interventions include the tasks that the system presents and the ways the system responds to input in the service of accomplishing that task. Interventions create the link between the embodied aspects of the system and spatial cognition by creating relationships between body movement (embodied interactions) and the spatial aspects of the content.

Descriptions of the interventions are drawn from our analysis of existing TEIs and are an attempt at a high-level description of what the user does with the system. As with the descriptions of embodiment, the column cannot include an exhaustive list since new systems will constantly create new things for users to do.

Intervention and embodiment are tightly coupled. Whether designers start with an intervention or with a method for establishing embodiment in mind, each must be designed in a way that supports the other. For example, an intervention that requires a person to walk around a room would not work well with a system that establishes embodiment by grasping and moving objects. The system would need to be updated with either a method for tracking walking or an intervention that uses grasping. Although this conclusion seems obvious, the strong link between scale of embodiment and intervention has implications for the aspect of spatial cognition that can be engaged by any given system.

### Summary

The three elements this diagram brings together—embodiment, intervention, and spatial cognition—describe the aspects of TEI design that work together to create tangible and embodied interactive systems that engage spatial cognition. By defining these elements and the categories they contain and illustrating their relationships on a diagram, we outline a design space for TEIs that fit within the focus of our research. This diagram provides a starting point for designers interested in working in this space and researchers interested in using TEIs for spatial cognition research. The systems described in the following sections were selected as good examples of systems that leverage embodiment to engage spatial cognition. Our analysis led to the understanding that embodiment and spatial cognition are linked through intervention. Plotting these relationships on the diagram presented above leads to observable trends and opportunities for research in this space.

## System descriptions

To better understand the relationships between embodiment, intervention, and spatial cognition as they relate to the design of interactive systems, we analyzed several classic interactive systems that engage the body and spatial cognition. The systems presented here were selected to be representative of the range of ways in which TEI systems have engaged spatial cognition from an interaction perspective and does not constitute a comprehensive set of TEI systems that relate to spatial abilities. These systems engage the body at a particular scale (embodiment) and ask the user to perform some task (intervention). The combination of embodiment and intervention may engage a particular aspect of spatial cognition related to representation, perception, or navigation. We define each system using the language of the design space and then plot the systems on the design space diagram to highlight trends and opportunities for research in the fields of cognitive science and HCI.

### Figural

The systems described in this section engage the body at a figural scale, by asking people to manipulate physical or virtual objects to accomplish some task.

#### FoldIt

FoldIt was developed by Cooper et al. (2010) as a way to crowdsource the task of finding the correct conformation of protein molecules. The conformation, or shape, of a protein molecule determines how it is used in a biological system. However, the specific shape of a protein cannot be directly inferred from its chemical structure and computers on their own are not particularly good at determining these shapes. FoldIt supported the use of people’s innate spatial skills to solve this problem.

FoldIt presents a virtual, three-dimensional (3D) image of a protein molecule which is not yet in its correct shape and enables a user to alter the shape of the molecule using a mouse. It was released to the public as downloadable software and has, at this point, engaged more than 57,000 players and shown that humans perform better on protein-folding tasks than computers (Cooper et al., 2010).

FoldIt turns a microscopic protein molecule into a figural scale object, which makes it possible for users to apply small-scale spatial abilities to alter its shape and develop an understanding of the kinds of relationships in the molecule that determine its form. Specifically, it establishes embodiment through the use of a mouse, which acts as a virtual proxy for the user’s hands. The intervention presents a virtual representation of a physical object and updates the shape of the object based on the user’s interactions. The system enables users to apply mental rotation and scaling skills to solve the problem of finding the correct shape for the molecule.

#### URP

URP was developed by Underkoffler and Ishii (1999) at the MIT Media Lab. URP is a tabletop-based urban planning simulation tool that urban planners can use to understand the relationships between building location, time of day, shadows, and wind.

The system tracks the position and orientation of physical objects on the surface of the table and displays the results of a simulation based on the placement of the objects. The objects in URP include models of buildings and a clock for changing the time of day. A user grasps and moves the building objects to set their positions and turns the hands of the clock to change the time. The system responds by displaying wind vectors and shadows on the surface, allowing users to develop an understanding of how the placement of the buildings alters the properties of the environment.

URP establishes embodiment by providing physical objects that a user grabs and moves. The intervention asks users to move the buildings and responds by graphically displaying the results of the simulation it runs. Because the objects are small-scale models of the buildings and the graphical display is a scaled representation of the environment, URP engages scaling ability along with providing a tool for students to develop mental rotation skills as they related to building and environmental scale objects.

#### Topobo

Topobo, developed by Raffle, Parkes, and Ishii (2004), is a construction kit that can record and play back physical movements. Topobo consists of a set of passive and active blocks. The passive blocks snap together to build small animal-like models. The active blocks contain a motor and snap together with the passive blocks. When a user rotates blocks connected to an active block, the system remembers the direction, speed, and magnitude of the rotation. The rotations can then be played back by the system. By coordinating the rotations of several “limbs” connected to active blocks in an animal-like model, users can create walking sculptures. Creating a successful walk requires considering aspects of the model like balance and direction of forces (Raffle et al., 2004).

Topobo establishes embodiment by providing physical blocks that are assembled into a larger object. This object itself can be grasped and manipulated. The intervention asks users to create an animated object and plays back that animation in the physical object. Building the object engages skills related to assembly. Animating the object engages skills related to force and motion or mechanical reasoning.

### Vista

#### Projection mapping and the Mystery Spot

Projection mapping techniques align digital projections with boundaries and surfaces of physical objects, from building facades, to furniture and walls. For example, The 600 Years, developed by Dan Gregor and Amar Mulabegović in 2010, projects onto the façade of a clock tower in Prague to depict the interweaving of the history of the tower and the country. Using projection mapping techniques, the designers make the tower appear to crumble and rebuild, catch fire, turn inside out, and merge with the sky (TheMacula - The 600 years, Astronomical Clock Tower, Prague, n.d.). By changing the coloration and creating the appearance of movement along these physical features, projection mapping can cause objects to appear to have spatial properties that are physically impossible. For example, rooms can be made to look larger than they actually are and building facades can appear to rotate or grow and shrink.

The Mystery Spot is a tourist attraction outside of Santa Cruz, California (The Mystery Spot Official Website, n.d.). By constructing a cabin with walls that are perpendicular to a hillside, instead of vertical with respect to gravity, the Mystery Spot creates illusions where balls appear to roll up hill and short people appear taller than tall people. The way the Mystery Spot is constructed breaks the link between visual perception and the proprioceptive and vestibular systems causing objects and environments to appear to have spatial properties that do not make sense in the physical world.

While the Mystery Spot is not a digital, interactive system, it highlights ways that the body and perception of space are linked and illustrates techniques that designers of interactive systems might employ to create new ways to interact with vista scale spaces. By combining projection mapping techniques with embodied interactions, designers could create vista scale environments with spatial properties that change based on the bodies and movements that engage with them.

Ping Pong Plus is one early example of tracking objects and altering projections based on their movement (Ishii, Wisneski, Orbanes, Chun, & Paradiso, 1999). The Ping Pong Plus system uses projections to modify a standard game of ping pong. In one game, holes appear in the ping pong table wherever the ball bounces. Players lose the point if the ball hits an empty space (Ishii et al., 1999).

These systems would establish embodiment through the visual and vestibular systems and through walking around the rooms and interacting with objects and spaces in the room. Interventions could ask users to alter spatial properties to accomplish some task and would engage aspects of spatial cognition related to the perception of angles, distances, and directions that are constructed based on the state of the body.

#### SMSlingShot

SMSlingshot is an interface intended to create a sense of agency in public spaces by enabling people to write short messages and display them on the side of a building using an interface based on a slingshot. It was developed by Patrick Tobias Fischer, Thilo Hoffman, Sebastian Piatza, and Christian Zoellner in 2010 (Fischer, Hornecker, & Zoellner, 2013).

SMSlingShot is a physical slingshot augmented with a cell phone keypad. Users enter a message using the keypad then aim and shoot the slingshot at a distant wall. A projector then shines the message on the wall where the user aimed it. SMSlingShot does not necessarily alter the perceived spatial qualities of an environment, but it does illustrate an embodied interaction that enables people to interact with digital content in vista scale spaces (Fischer et al., 2013).

In SMSlingShot, typing the message and then aiming and drawing of the strings of the physical slingshot to send the message establishes embodiment. The intervention asks people to alter the visual qualities of a distant object. Depending on the content of the projections, a system like this could engage aspects of spatial cognition related to perception of distance and size.

### Environmental

#### Slower Speed of Light

Slower Speed of Light was developed by Gerd Kortemeyer, Philip Tan, and Steven Schirra in 2013 as a way to help physics students understand Einstein’s theory of special relativity (Kortemeyer et al., 2013). Slower Speed of Light is a video game played from a first person point of view in a navigable virtual environment. In Slower Speed of Light, players use a keyboard to move around a small environment collecting tokens. Each time a player collects a token, the speed of light in the virtual world slows down. As the speed of light slows down, the speed of the player’s movement and the speed of light converge. The game engine changes the visible qualities of the environment to show the effects of relativity, especially of moving nearly light speed.

Slower Speed of Light establishes embodiment using navigation of a virtual environment controlled by a keyboard. The intervention asks people to move through the environment and collect tokens and responds by altering the properties of the environment that relate to the theory of relativity. This system engages aspects of spatial cognition related to distance and heading estimation, construction of route and survey knowledge, and the relationship between distance and time.

#### feelSpace

The feelSpace belt was developed by Saskia K. Nagel and her collaborators at the Institute of Cognitive Science in Osnabrük, Germany to investigate the plasticity of the sensorimotor system by attempting to create a new sense (Riener & Ferscha, 2008). feelSpace is a belt with several vibration motors and a digital compass stitched into it. The motors are in contact with the person all around the person’s waist. When a person wears the belt and walks around, the vibration motor oriented towards the north constantly vibrates; when the person rotates, the motor that was oriented to the north and was vibrating turns off and the neighboring motor that is now oriented north starts to vibrate. Designers of the system asked users to wear the belt for several weeks and observed that, over time, people stopped noticing the vibration but were able to incorporate it into their sense of direction. In one study, blind participants who had worn the belt for an extended period of time were led around a complex path and asked to point back to the start of the path (Kärcher, Fenzlaff, Hartmann, Nagel, & König, 2012). Wearing the belt was shown to improve this particular spatial skill called homing.

feelSpace establishes embodiment through a coupling between the location of a constantly present physical sensation and the physical movement of the person through an environment. The intervention simply asks users to wear the belt as they go about their day-to-day lives. The system engages aspects of spatial cognition related to homing, heading recall, and the construction of survey and route knowledge.

## Discussion

Defining this design space and using it to describe existing TEIs leads to a more thorough understanding of how the body, action, and spatial cognition are related in different systems. Plotting these systems on the design space diagram provides a clear picture of the design trends and the design and research opportunities for embodied interfaces that engage spatial cognition.

### Populating the design space diagram

**Fig. 2:**
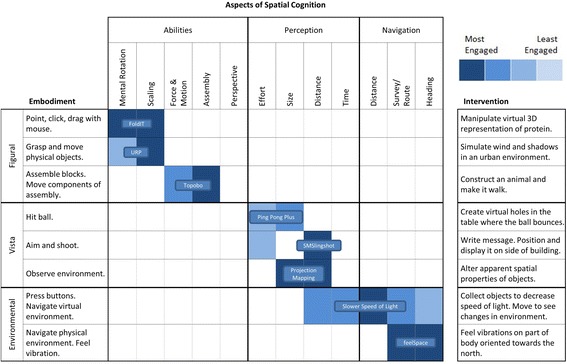
Plotting existing TEI systems on the design space diagram shows that different combinations of embodiment and intervention engage different aspects of spatial cognition

Figure 2 shows the result of plotting these systems with respect to the scale of their interaction and the aspect of spatial cognition they engage. The name of each of the systems described above is plotted on the diagram in relation to the way it engages the body and the aspect of spatial cognition its intervention appears to engage. Because none of these systems have been evaluated explicitly for their relationships to spatial cognition, the shades of blue highlight the aspects of spatial cognition a system is most likely to engage and how directly.

The intervention column is filled in with descriptions of the task that the system asks the user to perform. As a descriptive column, it serves as a reminder of what the names of the systems mean, but it is the systems, in their entireties, that support the relationship between the body and spatial cognition. This relationship is fully encompassed by the name of the system. The descriptions of the interventions are primarily a reminder of how the systems support that relationship.

Each system plotted in Fig. 2 incorporates relationships between embodiment and spatial cognition in different ways. Illustrating these relationships leads to insights about both design trends within the TEI community and opportunities for research in both TEI design and cognitive science. The trends and opportunities, which are described in detail in the following section, provide starting points for the design of new systems which can be evaluated from a spatial cognition perspective. The “Tangibles for Augmenting Spatial Cognition” project, discussed later in this article, illustrates how working within this design space can lead to interfaces that engage specific aspects of spatial cognition and how to evaluate that engagement.

### Trends and opportunities

**Fig. 3:**
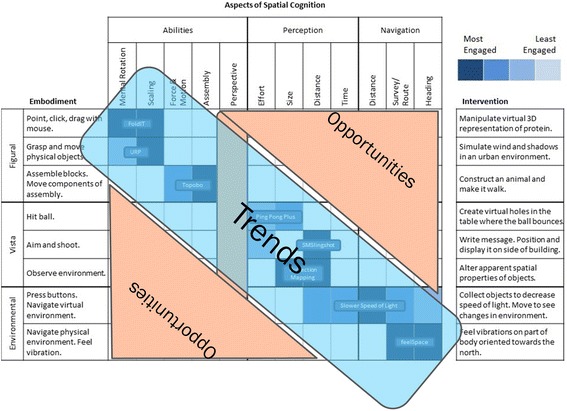
The populated design space diagram shows a trend in the relationship between the scale of embodiment of a system and the aspect of spatial cognition that system engages. It also makes it clear that there are opportunities for research and design of systems that create new relationships between the body and space

Figure 3 highlights the trends and opportunities for research in this design space:

The main trend highlighted by Fig. 3 is that the scale of interaction tends to relate to the aspect of spatial cognition a system engages. While some systems do cross the boundaries of the spatial cognition classifications, broadly speaking, figural scale embodiment relates to small-scale spatial abilities, vista scale embodiment relates to perceptual effects, and environmental embodiment relates to navigation abilities. This trend can give designers a starting point for either designing a system to target a specific aspect of spatial cognition or selecting an aspect of spatial cognition that a particular system may be well-suited to engage. The reasons for this trend are unclear, but it may be that simply selecting the right tool for the job (or the right job for the tool) led designers to the sweet spots that balance scale of embodiment with the different aspects of spatial cognition. If that is the case, then the gaps in the diagram reveal opportunities for research in this space.

First, there is an opportunity to design systems that more directly engage perspective-taking abilities. Our analysis did not find any TEI systems that engage perspective taking, even though it has been shown to be linked to the body (Tversky & Hard, 2009). Again, this could be related to the fact that, until recently, technology that engaged the body in a way that related well to perspective taking was cumbersome and expensive. Increased availability of virtual reality equipment may lead to additional systems that engage perspective-taking skills.

A second opportunity is for the development of systems that engage the body across multiple scales. Again, access to technology may be the limiting factor here. Large systems that alter the apparent properties of vista scale environments or that outfit environmental scale spaces with sensors and that respond to people’s movement through them have, up until recently, been expensive and complicated to develop. The increasing availability of technologies like Arduino microcontrollers, high-resolution and high-frame rate cameras, and brighter projectors with very high resolutions will continue to make building large and complex interactive systems more approachable. Combining these large-scale systems with tangible objects or virtual reality headsets can create systems that use figural scale interactions to engage vista and environmental scale aspects of spatial cognition, or that more readily translate large-scale data to figural scale interactive systems.

Finally, by focusing the design of systems specifically on engaging spatial cognition, designing interfaces that target a single spatial skill and that include controls that limit the use of additional skills can lead to new research opportunities in the cognitive and psychological sciences. In particular, as the case studies below will show, drawing inspiration from the cognitive sciences to help inform and shape how interfaces establish embodiment and how interventions target specific skills can lead to interfaces that can be evaluated using methods from cognitive science and psychology to show their specific effects on spatial cognition. Changing specific elements of the interface and intervention and evaluating the effect of those changes can lead to insights about how specific aspects of embodiment are related to spatial cognition.

### Expanding the design space

Our own projects, emBodied Digital Creativity (BDC) and Tangibles for Augmenting Spatial Cognition (TASC), illustrate cognition-focused design and show how it can lead to systems that create new experiences and support cognitive science research. Each of these projects expands the design space presented in this article by leveraging sensing and display technologies to create novel embodied experiences. These projects combine figural and vista scale methods for establishing embodiment, attempt to eliminate variables that might confound their effects on spatial cognition, and show how the use of spatial skill evaluations can lead to new insights about the relationship between the body, spatial cognition, and digital media.

#### BDC: emBodied Digital Creativity

**Fig. 4:**
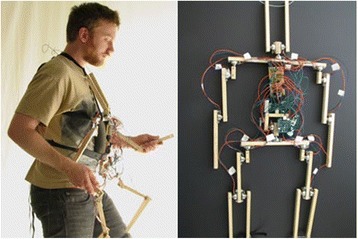
The BDC interface hangs from the shoulders and attaches at the knees in such a way that it moves with the person wearing it

BDC was a National Science Foundation-funded project that sought to develop a system that leveraged ideomotor coding to create a strong sense of identification with a virtual avatar and use that system to support creativity. BDC was developed in the Synaesthetic Media Lab at Georgia Tech between 2008 and 2011. The system creates a one-to-one mapping between the movements of a human-shaped puppet (Fig. 4) and a virtual 3D avatar. To use the system, a person wears the puppet between her shoulders and knees (Fig. 4) and holds on to the puppet’s arms. The puppet moves along with the person and generates the data necessary for rotating the joints of the avatar into the correct position.

Cognitive science research informed the project during all phases. The first phase of the project used a common coding approach to design, build, and evaluate an interface that mapped own body movement onto a virtual character to create a strong sense of identification with the avatar (Mazalek et al., 2009, 2010). During the second phase of the project, we developed and evaluated an intervention focused on engaging mental rotation ability because of its link to creative problem solving (Mazalek et al., 2011).

**Fig. 5:**
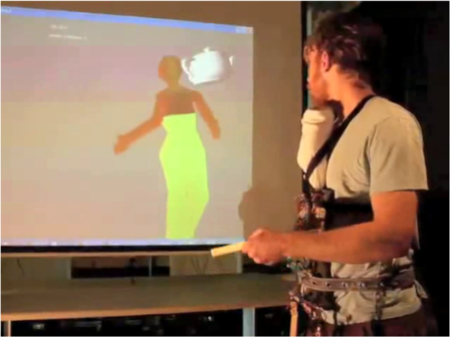
In the teapot touching intervention, the camera flies around the virtual avatar, continuously altering the spatial relationship between the avatar and player. The player must perform mental rotation to use the puppet controller to make the avatar touch teapots as they appear

The mental rotation intervention, shown in Fig. 5, asked players to use the puppet to make an avatar reach out and touch floating teapots while a virtual camera moved around the avatar. The camera movement caused the player to experience shifts in the spatial relationship between her physical and virtual bodies. This intervention forced players to continuously perform mental rotation in order to act in a constantly changing environment. We evaluated how the intervention influenced mental rotation skills by asking participants to complete a standard mental rotation test before and after playing the teapot game (Mazalek et al., 2011).

BDC expands the design space by establishing embodiment at both the figural and vista scales by leveraging ideomotor coding and physical feedback to create identification with a remote body. The intervention targeted mental rotation ability by creating an embodied experience that forces players to continuously perform mental rotation.

#### TASC: Tangibles for Augmenting Spatial Cognition

The TASC project is supported by a grant from the Canadian Social Sciences and Humanities Research Council and is part of ongoing research aimed at identifying the important design considerations for embodied interactive systems that support spatial abilities, specifically for STEM learning. The TASC intervention targets perspective taking because of the correlations between perspective taking and large-scale spatial cognition and map reading (Hegarty & Waller, 2004; Liben & Downs, 1993).

**Fig. 6:**
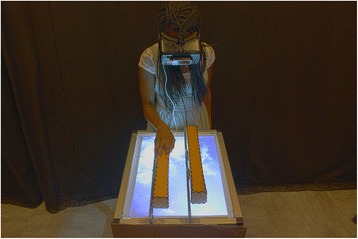
The TASC system establishes embodiment through a combination of virtual reality with head and hand tracking and physical feedback provided by the blocks, which can be grasped and moved

**Fig. 7:**
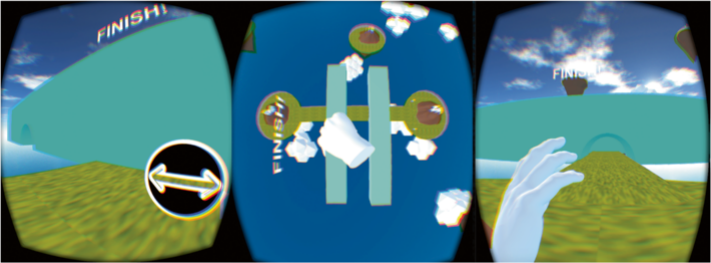
The TASC intervention asks players to align tunnels in virtual blocks to create a clear path between the player’s position in the virtual world and some goal. The player must work from multiple perspectives to determine the position of the tunnels and to move the blocks to the correct position

The TASC system (Fig. 6) combines a virtual reality headset that tracks head movements, a separate sensor that tracks hand movements, and a set of physical blocks that are tracked by the table beneath them. The player can look around the environment and see her hands as she reaches out, grabs, and moves the physical blocks. During the intervention (Fig. 7), a player standing on the ground sees a bridge which is obstructed by two large blocks. To the left or right, the player can see tunnels through the blocks. The player switches to a top-down view of the environment in order to grasp and move the blocks. The player must align the tunnels with the bridge using the information obtained from the ground-level view. This intervention forces the player to perform perspective taking in order to cross the bridge.

The system bridges figural and vista scale embodiment by combining multiple types of embodied interaction with an intervention that requires working from multiple points of view. The intervention specifically targets perspective taking, which had not been supported by previous systems. It is currently being tested using a digital version of the perspective-taking evaluation developed by Kozhevnikov and Hegarty (Hegarty et al., 2006; Kozhevnikov & Hegarty, 2001).

#### The expanded design space

**Fig. 8:**
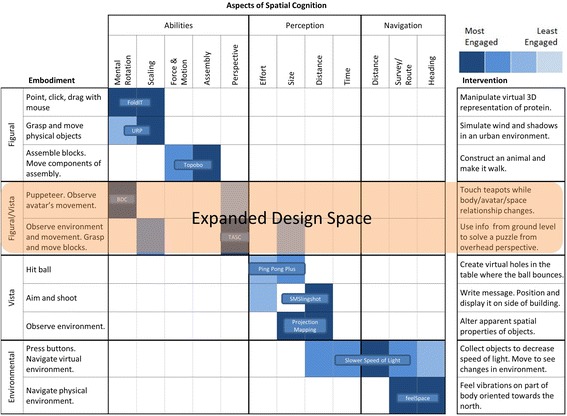
The BDC and TASC systems expand the design space by combining figural and vista scale embodiment and targeting perspective taking. BDC uses a figural scale puppet controller to manipulate a distant avatar. TASC uses virtual reality to enable a player to interact with a single environment at both the figural and vista scales

Plotting BDC and TASC on the design space diagram reveals how their focus on engaging spatial cognition leads to novel methods of establishing embodiment and expands the design space, as illustrated in Fig. 8.

By leveraging ideomotor coding to establish embodiment, BDC and TASC bridge the figural and vista scale interactions. Both display representations of own movement at a distance in such a way that performing a figural scale action is experienced as engaging with vista scale content. Both projects manipulate the spatial relationship between the physical body and the digital environment in order to engage spatial cognition. By incorporating multiple points of view and identification with remote bodies in to their design, each system engages perspective-taking skills, something that previous TEI systems have not accomplished. The following sections describe the challenges and opportunities we have uncovered during our work on BDC and TASC and that face researchers working in this design space.

## Conclusion and further considerations

Given the strong link between the body and spatial cognition, TEIs are particularly well suited to engage, support, or alter different spatial skills. This paper presents a design space that highlights the ways that current systems exploit the link between body, action, and space to achieve their goals. Our own work on BDC and TASC illustrate how this design space definition can be useful for creating systems that intentionally engage particular aspects of spatial cognition. Evaluating these systems for their effect on spatial skills provides new understanding of ways to support spatial skills as well as the ways those spatial skills are related to the body. This research aims to eventually lead to new interventions in educational contexts and has impacted the field of HCI and the cognitive sciences.

### Broader impacts

Our research makes contributions to the field of HCI and the cognitive sciences. By applying research showing that cognition is embodied to the design of interactive systems and evaluating them to show that engaging the body can support cognition, our research has led to a new approach to HCI. This approach is novel in both the interaction methods it employs and the goals of the systems that it produces. Traditional HCI aims to develop systems that make completing a task as efficient as possible. By focusing on supporting cognition, our systems do not necessarily make solving problems easier or faster, but they create new ways for people to think about the problems they solve, which may lead to greater transferability of problem-solving skills across situations. The interventions that these systems include do not necessarily help people do work. Instead they create situations in which skills can be developed that support work in different contexts.

Evaluating these systems using methods from the cognitive sciences has already shown that embodied interaction can lead to a sense of identification with digital content (Mazalek et al., 2009, 2010) and that this identification can be leveraged to improve mental rotation ability (Mazalek et al., 2011). Future work in this space will refine our understanding of how the body and cognition relate and eventually lead to systems that more effectively exploit these relationships in support of different system goals.

### Future work

Our future work in the space of TEI and spatial cognition will incorporate two threads: refining the framework and design space presented in this paper by designing and evaluating new systems and integrating our research with educational practice with user-centered design practices.

#### Framework and design space

The design space presented in this paper and the design framework that it establishes lead to new ways to think about the design of TEI systems. The idea that TEIs can engage specific aspects of spatial cognition through embodiment and intervention leads to a refined sense of the impact of design decisions on user experience and cognition. Conversely, the clarification of how, through design, TEIs can leverage specific spatial skills to support learning, work, and task accomplishment can lead to novel interactive systems.

In order to refine the design space and increase its utility, we will conduct the following future work. First, we will support the design space definition with fundamental research. Evaluating the TASC system to determine its effect on perspective-taking skills and to determine the specific design elements that create that effect will give us a more nuanced understanding of the effect of design decisions on a system’s relationship to spatial cognition. Based on our fundamental research and discussions with the research community, we will refine the design space so that it can support new research in both cognitive science and HCI. From our refined design space, we will develop and test new TEIs that incorporate aspects of design that best engage spatial cognition. These iterative phases of designing, building, and testing TEIs will lead to continued refinement of the design space as well as produce new knowledge related to cognition of space.

#### Practical integration with education practice

Given recent research linking movement, comprehension and retention (Kontra, Lyons, Fischer, & Beilock, 2015), and body and action to spatial cognition, the physical movements supported by TEIs may provide opportunities to incorporate physical movements into curricula. Ultimately, understanding the ways that TEIs can engage spatial cognition can lead to approaches to improving spatial cognition that target STEM education. But before that can happen, there are countless challenges to making changes to curricula in both formal and informal learning environments, e.g. cost, time, and government standards. Therefore, education practices, tools, and curricular requirements must be considered all together when developing ways to systematically incorporate spatial skills into education. Determining the best approach to meeting these challenges will require feedback from all stakeholders in order to understand their needs and include them in the design and implementation of new tools and practices. Early phases of moving our work towards educational practice have included gathering feedback and information from educators about the challenges and opportunities associated with targeting spatial skills and incorporating new technologies in educational practices. Over the course of this project, we will host workshops and interview stakeholders within education systems. Our findings from these workshops and interviews will be incorporated in the to the design framework so that it provides a way to discuss, not only the relationship between body, space, and action, but also a particular design’s utility for education.

As we continue to refine our designs and the framework, we aim to identify opportunities to work directly with educators and students to design, develop, implement, and test a system that functions, from a holistic/systemic point of view, to support, alter, and improve spatial cognition in high-priority populations.
